# Chronic Milk-Dependent Food Protein-Induced Enterocolitis Syndrome in Children from West Pomerania Region

**DOI:** 10.3390/nu13114137

**Published:** 2021-11-19

**Authors:** Karolina Bulsa, Małgorzata Standowicz, Elżbieta Baryła-Pankiewicz, Grażyna Czaja-Bulsa

**Affiliations:** 1 Szczecin Outpatient Clinic, 71-050 Szczecin, Poland; k.bulsa@gmail.com; 2 Doctoral Studies, Pomeranian Medical University, 70-204 Szczecin, Poland; malgo.kaczorowska@gmail.com; 3 Neonatology Clinic, Pomeranian Medical University, 70-204 Police, Poland; elzpan@gmail.com; 4 Chair and Department of Paediatrics and Paediatric Nursing, Pomeranian Medical University, 70-204 Szczecin, Poland

**Keywords:** milk allergy, children, non-IgE mediated CMA, food protein-induced enterocolitis syndrome, FPIES

## Abstract

Characteristics of chronic milk-dependent food protein-induced enterocolitis syndrome (FPIES) in children from the region of Western Pomerania were studied. Prospectively, 55 children were diagnosed at a median of 2.2 months. The open food challenges (OFC), morphologies, milk-specific IgE (sIgE) (FEIA method, CAP system), and skin prick tests (SPTs) were examined. Vomiting and diarrhea escalated gradually but quickly led to growth retardation. Of the infants, 49% had BMI < 10 c, 20% BMI < 3 c; 25% had anemia, and 15% had hypoalbuminemia. During the OFCs we observed acute symptoms that appeared after 2–3 h: vomiting diarrhea and pallor. A total of 42% children required intravenous hydration. Casein hydrolysates or amino acids formulae (20%) were used in treatment. In 25% of children, SPT and milk sIgE were found, in 18%—other food SPTs, and in 14% allergy to other foods. A transition to IgE-dependent milk allergy was seen in 3 children. In the twelfth month of life, 62% of children had tolerance to milk, and in the twenty-fifth month—87%. Conclusions. Chronic milk-dependent FPIES resolves in most children. By the age of 2 children are at risk of multiple food sensitization, and those who have milk sIgE are at risk to transition to IgE-mediated milk allergy. Every OFC needs to be supervised due to possible severe reactions.

## 1. Introduction

Cow’s milk allergy (CMA) is the most common allergy in the first year of life. It takes two forms: the IgE-mediated CMA (IgE-CMA) and the non-IgE mediated CMA (non-IgE-CMA). Non-IgE-CMA is characterized by digestive symptoms and has a good prognosis, usually resolving before the age of three. In contrast to IgE-CMA, the diagnosis of various non-IgE-CMA syndromes can be challenging due to the overall lack of non-invasive confirmatory testing for these disorders. Many of the non-IgE-CMA syndromes are diagnosed clinically based on history, diagnostic milk-free diet, and followed by positive milk provocation test, which is a “gold standard” for diagnosing these diseases [1,2].

The first classification of non-IgE-CMA gastrointestinal disorders was proposed by Sampson HA in 2003 [3]. The classification was adopted by WAO in 2010 (DRACMA) and EAACI in 2014. [4,5]. It covers three items: food protein-induced enterocolitis syndrome (FPIES), food protein-induced allergic proctocolitis (FPIAP), and food protein-induced enteropathy syndrome (FPIE) as well as the syndrome of eosinophilic gastrointestinal diseases (EGID), where milk can cause allergic reactions under the IgE-dependent and IgE-independent mechanism.

FPIES is a non-IgE cell-mediated food allergy [6]. The first international consensus on these diseases was published in 2017 by an international workgroup convened through the Adverse Reactions to Foods Committee of the AAAAI and the International FPIES Association advocacy group [7]. Previously, diagnosis had been made based on descriptions [8,9]. 

FPIES usually manifests with repeated vomiting, and less commonly with watery diarrhea, often accompanied by lethargy and pallor. Severe cases can lead to dehydration with ionic disturbances, acidosis, methemoglobinemia, and hypotension (in at least 15% of reactions) mimicking sepsis. Delayed onset (1–4 h after food ingestion) and absence of cutaneous and respiratory symptoms suggest a systemic reaction different from anaphylaxis [7].

FPIES is a syndrome that occurs in two forms, acute and chronic. The acute form of FPIES is much more severe and is caused by food ingested intermittently or after a period of avoidance (solid foods); therefore, it occurs in infants no sooner than after the introduction of modified diet, i.e., usually after 6 months of life. The foods that most commonly cause acute FPIES are rice and oat, which account for almost ^1^/_3_ of cases in the USA and Australia [9,10,11]. In 2009, Mehr et al. highlighted the emerging importance of rice, a food commonly thought to be “hypoallergenic”, which cause severe FPIES [11]. In Spain and Italy, FPIES is often caused by a fish-based diet, which is rare in other countries [7,12,13]. Other foods more likely to cause FPIES symptoms include corn, peas, poultry, egg, and goat milk [7].

The chronic form of FPIES is caused by regularly administered food, typically milk or soy infant formula. It is reported only in infants younger than 4 months of age, usually shortly after the end of breastfeeding (2–3 weeks). The main symptoms of chronic FPIES are intermittent vomiting and watery diarrhea rapidly leading to weight and growth deficits. Severe type of chronic FPIES can lead to dehydration and hypoalbuminemia. During the food oral challenge an acute reaction always occurs in the following order: first vomiting (after 1–4 h of food ingestion) followed by watery diarrhea (after 5–10 h of food ingestion). This acute symptomatology during the oral challenge after food avoidance is typical for chronical FPIES. It is also the basis for distinguishing chronic FPIES from FPIE and eosinophilic gastroenteritis [1,7]. 

The purpose of the study was to describe chronic milk-dependent FPIES (chronic milk-FPIES) in children from the region of Western Pomerania who were diagnosed over a 5-year period (2014–2018).

## 2. Materials and Methods

The prospective study was conducted for 7 years (2014–2020). During the first 5 years (2014–2018), chronic milk-FPIES was diagnosed in 57 children. They lived in the region of West Pomerania and were patients of the Paediatric Gastroenterology and Rheumatology Clinic, Gastroenterology Outpatient Clinic or Allergy Outpatient Clinics in Szczecin.

In the last 2 years of study (2019–2020) we continued the observation of the study group and we did not include new patients. 

**Figure 1:**
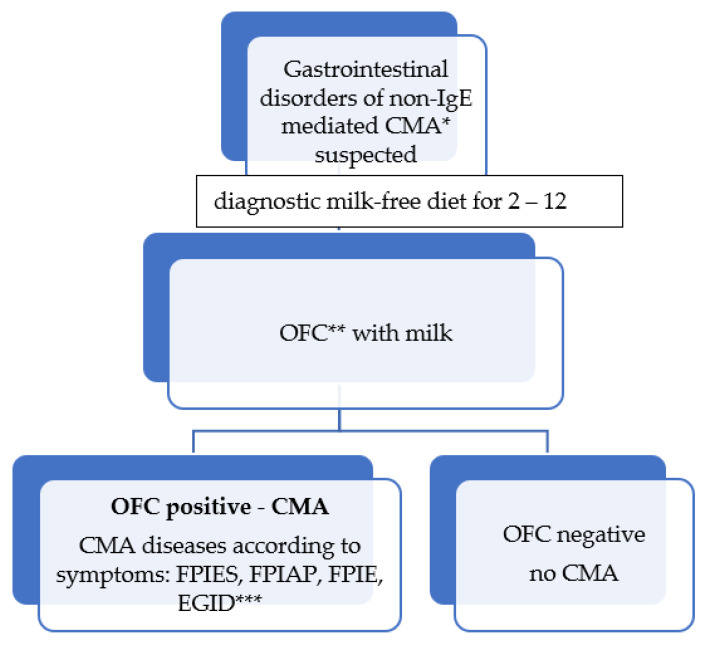
Gastrointestinal disorders of non-IgE mediated CMA diagnosis scheme * CMA—cow’s milk allergy; ** OFC—Oral Food Challenge; *** CMA diseases according to symptoms: FPIES—food protein-induced enterocolitis syndrome, FPIAP—food protein-induced proctocolitis, FPIE—food protein-induced enteropathy syndrome, EGID—syndromes of eosinophilic gastrointestinal diseases.

The patients were selected from children with symptoms indicating CMA. The suspicion of CMA was the premise for including a milk-free diet for 2–12 weeks, depending on the symptoms (Figure 1). After the symptoms had resolved or decreased, the milk oral food challenge (OFC) was performed. A negative OFC outcome ruled out CMA. A positive OFC was the basis for CMA recognition. FPIES was diagnosed in these children, with a delayed response during OFC (symptoms occurred above 2 h after milk ingestion), in whom occurred vomiting, pallor, and diarrhea, and often also severe dehydration.

The criteria for including a child in the study were: chronic milk-FPIES, age (up to 4 months), absence of coexisting chronic diseases, and a parental/legal guardian’s written consent for the child to participate in the controlled study. Consent also included the storage and publication of the collected data. Only 55 children (33 boys, 60.0%) at the age of 1.6–2.6 months (median 2.2 months) were included in the study. After 19 months of treatment, three patients discontinued their participation in the project (they did not report for control milk provocations). Ultimately, 52 children (94.5%) completed the study.

Each patient had a medical and allergic history (recorded recurrent adverse reactions) and underwent physical examination. Every 6 months medical examination was carried out and a follow-up milk OFC was performed. If adverse symptoms occurred after milk ingestion, the provocation was stopped. A positive OFC outcome affirmed the persistence of CMA and was a premise for continuing a milk-free diet. A negative OFC indicated that the child had developed tolerance to milk. 

**Figure 2:**
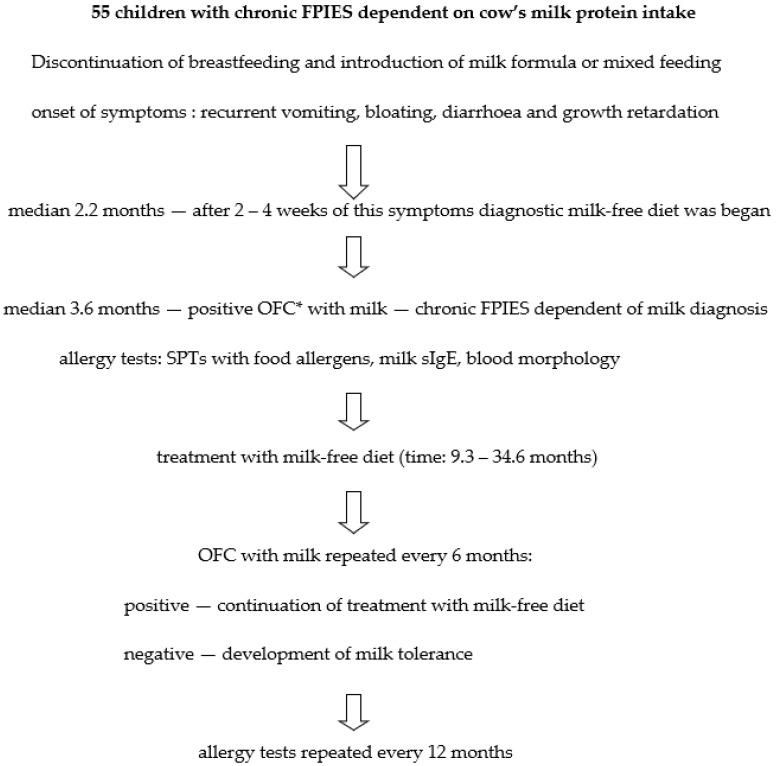
Diagnostic and treatment scheme in the study group of children with chronic FPIES dependent on cow’s milk protein intake. OFC *—Oral Food Challenge.

At the time of chronic milk-FPIES diagnosis and during follow-up visits (usually once per year, during the OFC procedure) blood morphology and cow’s milk-specific IgE (sIgE) concentration in serum were tested. In addition, skin prick tests (SPTs) with food allergens were performed (Figure 2).

During the diagnostic elimination diet and in the treatment of chronic milk-FPIES, a milk-free diet was administered: either the milk of mothers remaining on a milk-free diet or extensively hydrolyzed infant formulae (eHF; lactose-free casein hydrolysate). Children diagnosed with severe milk allergy received free amino acids formulae (AAF). 

The age of chronic milk-FPIES diagnosis was also the age of introduction of a diagnostic milk-free diet. A severe form of CMA was diagnosed according to WAO and ESPGHAN recommendations [4,14]. In all children the diagnosis of FPIES followed the 2017 criteria [7]. Previously, we used the Sicherer et al. criteria [8].

The open OFC procedures were always commenced in hospital conditions, under the control of a nurse and a doctor, with access to anti-shock drugs [15]. After a negative lip test (a drop of milk), gradually higher doses of milk were administered every 15 min (1, 2, 5, 10, 20, 50, and 100 mL). Infants younger than 6 months received at least 100 mL of milk. Patients remained under observation for at least 4–8 h following the end of OFC [15,16,17,18]. The provocation was continued at home for the next 6 days. Every day, parents administered the milk mixture corresponding in volume to one meal (older children—up to 250 mL), the information about possible adverse reactions was recorded in the observation card. After 6 days (or earlier, if side effects had occurred), the doctor examined the OFC outcome. In total, the results of 139 milk OFCs were analyzed. 

During the first OFC all the children had an intravenous entry, while during the next OFC, only those with elevated milk sIgE levels.

Cow’s milk sIgE concentration was determined by FEIA method in CAP system with automatic UniCAP apparatus from Phadia. The determination parameters were the range between 0.35–100 kU/L; accuracy between 2–9.1%; sensitivity < 2 kU/L; repeatability at 98%; specificity at 100%. Recommended range: healthy patients < 0.35 kU/L, atopics > 0.35 kU/L.

Skin prick tests (SPTs) with food allergens were performed with a modified method on the skin on the patient’s back. The positive control was histamine solution (1 mg/1 mL), while the negative one—a diluent. Commercial Allergopharma and ALK food solutions were used. The wheal and erythema size were measured after 20 min. SPT was regarded positive when the sum of the half of the longest diameter and the midpoint orthogonal diameter of the wheal with allergen was at least equal in diameter to the histamine wheal and at least 3 mm higher than the negative control.

Finally, all data were collected in electronic form in MS Excel spreadsheet and were subject to statistical analysis. Continuous variables were described by median, minimum, and maximum values. Discrete variables were described by their abundance and frequency of occurrence.

The research was approved by the PUM Bioethics Committee No KB-0012/80/14. The research was financed by the statutory activities (WNoZ-319-01/s/12/2013–2020) and by the NCN grant No 2016/21/N/NZ7/03409. The presented results are part of the ongoing project.

## 3. Results

Chronic FPIES after consumption of cow’s milk proteins was diagnosed in 55 children in median age of 2.2 months (1.6–2.6 months) (Table 1). It was the age at which the diagnostic milk-free diet began. Symptoms of CMA appeared after cessation of breastfeeding and introduction of milk formula (80% of children) or mixed feeding (20% of children). After 2–4 weeks of CMA symptoms, a milk-free diet was started. Vomiting and diarrhea escalated gradually but quickly led to physical retardation. 

At the time of diagnosis as many as half of infants (49%) had low BMI < 10 c (Table 1). In every fifth child it was extremely low at BMI ≤ 3 c. A quarter of the children had iron deficiency anemia and 15% of the children suffered from hypoalbuminemia. 

All 55 children with chronic milk-FPIES were affected by the recurrent vomiting, bloating, and diarrhea, sometimes with mucous (42%) and blood in stools (31%) (Table 1). Their weight gain was poor. 

The first milk OFC (1. OFC) was performed in children in median age of 3.6 months (2.1–5.5 months). Vomiting (after 2–3 h), diarrhea (after 4–10 h), and pallor were observed in all children (Table 1). As many as 42% of children required intravenous hydration and 7% received ondansetron. Their blood tests revealed increased white blood cell count and neutrophilia in 80% of children. There was no eosinophilia. No child had acidosis or methemoglobinemia. After the diagnosis of chronic milk-FPIES was made, parents were informed that the supply of milk or milk products to their children should only be attempted under medical supervision. The supply of these foods at home can be dangerous.

A family history of atopy was reported in 64% of children (mother—52%, father—58% or siblings—29%) (Table 1). At the time of diagnosis of chronic milk-FPIES, 18% of the children also had atopic dermatitis (AD). 

Lactose-free casein hydrolysates (eHF) and AFF were used in treatment of children with chronic milk-FPIES. AFF was administered to 11 children (20%) who were diagnosed with severe CMA type (BMI ≤ 3 c). Resolution of allergy symptoms and improvement in health were observed in all infants already after 3–14 days of dietary treatment.

In every fourth child the elevated sIgE and positive SPT for cow’s milk proteins were found (Table 1). Typically, sIgE values were low, in the range of 0.35–0.7 kU/L, rarely higher (2.8, 3.9, 6.9, 28.3 kU/L) and they decreased in the next tests. At the time of chronic milk-FPIES diagnosis no immediate reactions after milk consumption were observed. Immediate symptoms after milk ingestion (IgE-dependent CMA) were seen in 3 children (5.5%) during the subsequent challenge trials (at 19, 25, and 26 months of age) (Table 2). During the provocation, those children developed extensive urticaria, with two of them also suffering from bronchospasm. At that time no symptoms of chronic milk-FPIES were seen in those children. In the following years, their milk sIgE levels increased. Due to lack of parental consent, we could not perform control provocations during 3 years of observation.

Apart from allergy to milk, 10 children (18%) had positive results of SPTs to other foods (egg white, wheat, corn, banana, soybean, peanut), in some cases up to 4 positive SPTs in one child (Table 1). When these foods were first introduced to the diet, two infants developed symptoms of IgE-dependent allergies. One child presented mild urticaria about 30 min after egg white ingestion (at 8 months of age), while second (after 6 months of age)—after wheat intake (Table 3).

### 3.1. Introducing New Foods into Infants’ Diet

According to existing recommendations, parents were suggested to expand the infants’ diet after 4 months (2008: EAACI, AAP, 2009—ESPGHAN, 2010—NIAID). Most parents did so (89%), while others began introducing new foods after their babies were 5 months old (Table 4).

All new foods were given at home by parents for 4 days, at a dose consistent with typical intake. The absence of an adverse reaction was the basis for recognizing tolerance to that food and introducing it permanently into the diet [19]. If there were adverse reactions, allergy was diagnosed after contact with a doctor. As the reactions were not severe, the parents repeated the administration of these foods after a few days to check its reproducibility.

The order of products introduced into the diet was changed as recommended by other researchers [8,20]. All parents used a modified dietary expansion method with their children. Pumpkin, broccoli, and cauliflower were served as first foods in a form of a watery mush (Table 4), followed by carrots, potatoes, green beans, zucchini, beets, and parsley. Vegetables with a high risk of causing an allergic reaction, such as sweet potatoes and green peas, were not given until after 6 months of age. 

After 5 months of age, the diet was expanded by fruits such as peach, grapes, avocado, watermelon, and blueberries. Fruits more frequently causing allergic reactions, i.e., apple, pear, banana, and strawberries were introduced to the diet no sooner than after 6 months of age.

The greatest problems occurred with the supply of cereals, which are given at this age in a form of gruel or porridge. After 6 months of age, the supply of corn and wheat was suggested. Cereals, which in these children most often cause allergy symptoms, i.e., rice and oats, were introduced to the diet between 8 and 10 months of age.

Meat, fish, and egg were given at the same time as is recommended for healthy infants (Table 4). The order of supplying meat types was changed. Rabbit meat was recommended as the first meat, followed by pork and turkey meat. Beef and chicken were not introduced to the diet until the eighth month of age. Soya was not recommended until the twelfth month of age, nor was goat milk because of its high homology to cow’s milk.

During the introduction of new foods into the infants’ diet, allergy symptoms of IgE-independent allergy were observed (in response to some of the administered foods, despite the delayed time of their administration) (Table 3). Those were skin lesions and/or loose stools occurring from 8 to 24 h after consumption of the harmful food. They occurred in 6 children (11%) in response to 6 foods. The most common foods were: apple (3.6%), rice (3.6%), chicken, and turkey meat (1.8% each).

### 3.2. Course of Chronic Milk-FPIES

The children were observed from 2 to 6 years of age. The weight deficiency present at the time of chronic milk-FPIES diagnosis regressed in all children by the twelfth month of age. Most of them reached a weight and height range of 25–75 c (48 children, 87%). Hypoproteinemia resolved within 2 months, and the treatment of iron deficiency anemia was usually completed before 6 months of age. Blood count abnormalities resolved within 1–2 months.

The chronic milk-FPIES symptoms in the twelfth month of life were absent in 62% of children, in the nineteenth month—in 78% of children (Table 2). The median duration of treatment with a milk-free diet was 16.8 months (range: 9.3–34.6 mo). After 18 months of observation the three of patients discontinued their participation in the project (they did not report for control milk provocations). After 25 months of age, chronic milk-FPIES symptoms persisted only in one child. They disappeared at the thirty-seventh month of age. 

During the 3. OFC and 4. OFC three children showed symptoms of IgE-CMA (Table 2). It was the transition of chronic-milk-FPIES to IgE-CMA.

In the next 2–4 years of follow-up, symptoms of IgE-mediated allergy were found following the ingestion of soya (1 child) and peanuts (2 children) (Table 3). All of them developed extensive urticaria.

At the time of diagnosis, 10 children (18%) had mild atopic dermatitis (AD) (Table 2). By the age of 2, it had resolved in 3 children and by the third year in another 2 children (50%). In the following years, 4 children (7%) developed hay fever and 3 children (5%) developed bronchial asthma (house dust mite allergy).

## 4. Discussion

FPIES was recognized and formally defined in the mid-1970s [21]. Until recently, both acute and chronic FPIES was considered very rare or underdiagnosed [22,23] due to symptoms that can easily be confused with other diseases, especially with sepsis in the instance of acute FPIES [1,7,20]. Intensive research in recent years has shown that FPIES is much more common than it seemed. Studies conducted in centers in Israel and Spain indicated that the cumulative incidence of FPIES in the birth cohort ranges from 0.34% to 0.7% [24,25]. Population studies in the USA have shown that physicians diagnose FPIES in 0.28% of children (<1 and 0.11% of infants) and in 0.22% of adults [26]. Obviously, the data regard two forms of the syndrome and its incidence not only after consumption of milk but of other foods as well.

In the group under study the first symptoms of chronic milk-FPIES occurred shortly after breastfeeding (Table 1). Most mothers decided to continue breastfeeding for a short time, i.e., one month. After 2–4 weeks of infant formula administration, vomiting and diarrhea occurred. The median age of chronic milk-FPIES diagnosis and of the initiation of milk-free diet were low—2.2 months. What is typical for FPIES induced by cow’s milk is the appearance of symptoms during formula feeding. Only a few cases of this syndrome have been described in breastfed babies when the allergens were transported with breast milk [27,28]. Researchers in Japan report FPIES in 10% of breastfed babies [29].

The major symptoms of chronic milk-FPIES are intermittent vomiting and watery diarrhea that rapidly lead to weight and growth deficits. In the study group, as many as half of infants had low BMI < 10 c, and in every fifth child the index was extremely low at BMI ≤ 3 c (Table 1). 

Although FPIES is a form of IgE-independent food allergy, patients often have atopy, including atopic dermatitis and/or food IgE sensitization. In the study group of children, AD was diagnosed in 18% of children (Table 2). Studies report more frequent AD co-association (31% to 57%) in the United States and Australia while in Korea, Israel, and Italy it is rarer (up to 9%) [7].

In chronic milk-FPIES, food IgE sensitization defined as positive food SPT or serum food sIgE levels occurs in from 4% to 30% of patients [5,7]. In the study group, positive milk SPT and serum milk-sIgE were found in a quarter of children (Table 1). Only in 4 children did the sIgE concentration for milk exceed 0.7 kU/L. Nevertheless, during the 1. OFC in none of them immediate reactions after milk supply were observed. The milk-sIgE concentrations decreased systematically with age. FPIES caused by milk for which there is an elevated sIgE concentration is called atypical FPIES [7,8]. FPIES caused by milk in a child without the presence of milk sIgE is called classical FPIES.

Caubet et al. reported that children with chronic milk-FPIES who had increased milk sIgE were more likely to have persistence of chronic milk-FPIES after 3 years of age compared with those without sIgE. [9]. We did not observe this phenomenon in the study group of children. We also found that the presence of allergies to other foods did not delay the development of milk tolerance.

Only 3 children with elevated milk sIgE levels developed symptoms of IgE-mediated milk allergy during subsequent challenge (Table 2). Thus, a transition of FPIES—IgE-independent milk allergy into IgE-mediated allergy was observed in these children. This is a typical phenomenon in children with FPIES and does not occur in other forms of non-IgE-mediated food allergy. In the study group, we observed such a reaction in 3 children during the third and fourth OFC in 18, 25, and 26 month-olds. During OFC (18–48 mL of milk), all of them developed extensive urticaria. Two of them also developed also bronchospasms (Table 2). In the following years, their sIgE of milk and casein were elevated. Over the 4 years of observation, we did not perform any provocation tests waiting for the period when milk sIgE would start decreasing.

Atopic sensitization to foods other than milk was observed less frequently (25% vs. 18%) (Table 1). Those foods were well tolerated by most children. However, two of them developed symptoms of IgE-mediated allergy (mild urticaria) already in infancy (wheat, egg white) (Table 3). In the following years, extensive urticaria occurred for two more foods—peanuts and soya (Table 3). The coexisting IgE-mediated food allergy at presentation or on follow-up assessment was also reported by other researchers in 20% to 40% of patients [9,11,12]. Children with FPIES are also more likely to be allergic (2–20%) to foods that typically cause FPIES. Cereals (rice, oat), vegetables (sweet potato, green beans, peas), and poultry meats (chicken and turkey), which are typically treated as a potential weak allergen, must be considered in the follow-up of FPIES, particularly in infants with FPIES to cow’s milk or soy. Infants with FPIES are at risk of developing hypersensitivity to many food proteins [7,9,24]. In the study group of children, during the introduction of new foods, symptoms of IgE-independent allergy to 6 foods were observed in 6 infants (11% of children). These were rice, apple, turkey, and chicken meat.

Due to significant weight loss, a one-fifth of FPIES patients met the criteria for severe form of CMA and were treated with an elementary diet. The others received lactose-free casein hydrolysates (Table 1). Milk and baked egg were not used during treatment of chronic milk-FPIES primarily because of the short duration of the disease (at median 25 months of age 87% children had already developed tolerance to milk) and the lack of such recommendations in non-atopic allergies [1,7]. 

For infants with chronic milk-FPIES, avoidance of all forms of milk, including baked and processed milk, is recommended due to a lack of sufficient studies, although tolerance to baked cow’s milk has been reported in a small case series [12,20,30]. Some researchers report that some infants with FPIES in OFC tolerate even more than 100 mL of cow’s milk [24]. Most researchers believe that the tolerable dose is low [7].

In infants it is recommended to continue breastfeeding. In the study group only 13% of children were breastfed for 6 months, then received eHF. 

The introduction of soy milk is not recommended because of the frequent co-occurrence of allergies to both these foods (in about 20–40% of US patients). This coincidence was not observed in patients from other countries such as Italy, Australia, or Israel [12,24,31]. In the study group of children symptoms of allergy to soya occurred only in 1 child in the second year of life in the form of IgE-dependent allergy (urticarial) (Table 3).

Goat and sheep milk are also not recommended in patients with chronic-milk-FPIES due to high homology of the protein sequences in these milks to cow’s milk [32].

As regards patients with FPIES, any modification of their diet requires strict supervision, especially when it involves the introduction of grains (rice, oats), poultry and legumes, as they often cause symptoms of acute FPIES, as described in the literature [7,11]. According to the recommendations, in the presented group of children those foods were administered after 8 months of age (Table 4). Simultaneously, meat and fish was included as well. The order of the types of meat to be introduced was changed. Rabbit meat was given first, followed by pork and then turkey and chicken meat and beef. Eggs and pork were given at the same time as it is recommended for children without FPIES (7 and 8 months of age) [1,7]. 

Despite the delayed time of introducing new foods into the infants’ diet, we observed IgE-independent allergy symptoms to 6 foods in 6 children (11%) (Table 3). They were skin lesions or loose stools occurring from 8 to 24 h after consumption of these foods: rice, apple, chicken, and turkey meat. No infant developed FPIES dependent on solid foods. 

Although children with chronic milk-FPIES are more likely to react to solid food, most commonly rice or oat, current feeding guidelines do not recommend delay in introducing complementary foods past 6 months of life due to FPIES [5,33,34]. It is recommended that parents introduce a new food as a single ingredient and wait at least 4 days before introducing another food to observe for the development of a reaction [19].

Delayed intake of these foods is more likely to trigger allergy symptoms [5,14]. It is believed that when an infant tolerates the first few foods introduced, dietary expansion may be more tolerable [7]. The use of an elimination diet is always associated with a risk of nutritional deficiencies, so a dietary consultation is recommended [7,35],

OFCs performed in patients with FPIES are high-risk procedures and therefore require medical supervision. In patients with acute FPIES, intensive vomiting quickly leads to dehydration, acidosis, and consciousness disorders that require intensive intravenous treatment. In addition, in patients with chronic FPIES, the supply of harmful food after a period of elimination triggers acute symptoms (e.g., after the first elimination diet period). In all the FPIES patients with elevated milk sIgE levels, who have been treated with a milk-free diet, there is a risk of a sudden reaction during the follow-up OFC.

Therefore, in clinical practice, OFCs are only used in the initial diagnostic evaluation in cases when the patient’s history is not clear, symptoms persist despite the elimination of the potential trigger food, or the time course of symptoms is atypical [7]. In an instance of a typical history and improvement on a dairy-free diet, OFC is not performed because the risk of complications following OFC might outweigh its benefits. OFCs are necessary when there is a need to find out whether the child has already developed tolerance to the eliminated food. In chronic FPIES we perform them more often, on average every 6 months, because the disease recedes faster. In acute FPIES, which regresses more slowly, OFCs are performed less frequently, every 12–18 months.

During 1. OFC, vomiting, diarrhea, and pallor occurred in all children in the study group (Table 2). As many as 42% of children required intravenous hydration and 7% received ondansetron. In the next challenge, the percentage of children with diarrhea, pallor, and dehydration decreased. Vomiting was the predominant symptom (Table 1). Dehydration was less frequent and was treated with oral rehydration.

An increase in leukocytosis and neutrophils (>1500 cells/mL) was also observed during OFCs, as reported by other researchers [7,9,21]. 

During the 3-year observation, milk tolerance developed in 89% of children with chronic milk-FPIES. In the twenty-fifth month of life the proportion was 87%, while at 12 months of life it was recorded at 62% (Table 2). In three children chronic milk-FPIES transformed into IgE-dependent milk allergy. Three other children dropped out of the study after 18 months of follow-up. Similar results were obtained by Hwang at al [36]. Population studies in Israel have shown that 60% of children with cow’s milk-induced FPIES develop tolerance by age of 1 year, 75% by age of 2, and 85% by age of 3 [24]. The retrospective US studies revealed that the tolerance was reached by 35% of children by age of 2, 70% by age of 3, and 85% by age of 5 years [10]. The median age of tolerance was 6.7 years. The Korean research shows that tolerance may occur sooner, after 6 or 12 months [36].

## 5. Conclusions 

Chronic-milk-FPIES is a form of IgE-independent allergy. It is a rare syndrome, manifesting itself in the youngest infants after the introduction of milk formula. The symptoms, vomiting and diarrhea, quickly lead to severe growth retardation. Diagnosis is difficult due to the absence of eosinophilia in the blood tests, but the presence of leukocytosis and neutrophilia, which are indicative of infectious diseases. Treatment consists of a milk-free diet. Patients have multiple sensitizations to other foods, both in an IgE-dependent and IgE-independent mechanism. The foods that cause allergy symptoms are different in these two types of allergies. Furthermore, the transition of milk-induced FPIES to IgE-mediated milk allergy may occur in this disease. The occurrence of this transition in the course of OFC poses a risk of severe complications; therefore, milk provocation in these patients always requires intensive medical supervision.

## Figures and Tables

**Table 1:** Characteristics of children from the study group with chronic food protein-induced enterocolitis syndrome (FPIES) dependent on cow’s milk protein intake in the study group of children.

Characteristic	Number of Children (*n* = 55)
Age at onset (median/range)	2.2 months (1.6–2.6 months).
Feeding at the time of onset	Milk formula—80%, breast milk with milk formula—20% (mixed)
Atopic background:	64%: mother—52%, father—58%,
-family history of atopy	siblings—29%
-personal history of atopy	25%
FPIES symptoms:	
-vomiting	100%
-bloating	100%
-diarrhea/with mucous/with blood	100%/42%/31%
Body weight:	
BMI * < 10 c	49%
BMI ≤ 3 c	20%
Laboratory findings:	
-iron deficiency anemia	25%
-hypoproteinemia	15%
Allergy tests:	
-peripheral blood eosinophilia	Absent
-elevated sIgE and SPT ** for milk	25%
-positive SPTs for another foods	18%
1. OFC ***	
-time (median/range):	3.6 months (2.1–5.5 months)
Symptoms:	
-vomiting (2–3 h)	100%
-pallor (2–3 h)	100%
-diarrhea (4–10 h)	100%
-dehydration/intravenous hydration	42%
-ondansetron	7%
-leukocytosis with neutrophilia	80%
Treatment:	
-lactose-free casein hydrolyzates	80%
-amino acids formulae (elementary diet)	20%

* BMI—body mass index; c—percentile; ** SPT—skin prick test; *** OFC—oral food challenge.

**Table 2:** Natural history of chronic food protein-induced enterocolitis syndrome (FPIES) dependent on cow’s milk protein intake in the study group of children.

Characteristic	Number of Children (*n* = 55)
Age of tolerance to cow’s milk development (median/range):	
2. OFC *—12.2 months: 11.5–14.2 months	62%
3. OFC *—19.4 months: 17.4–20.8 months	78% **
4. OFC *—25.2 months: 24.5–26.5 months	87%
5. OFC *—31.1 months: 29.8–31.3 months	87%
6. OFC *—36.8 months: 36.2–37.2 months	89%
Treatment time on a milk-free diet to achieve tolerance (median/range) 16.8 months: 9.3–34.6 months	*n* = 49 children
FPIES transition to IgE-dependent milk allergy:	3 children (5.5%)
3. OFC *	1.8%
4. OFC *	3.6%
Symptoms in children transition to IgE-dependent milk allergy	
-extensive urticaria	5.5%
-bronchospasm	3.6%
Comorbidities:	
-Atopic dermatitis	improvement in half of children
-Hay fever	7%
-Asthma (house dust mite allergy)	5%
Symptoms during the next OFC *:	
-vomiting (2–3 h)	100%
-pallor (2–3 h)	less than 100%
-diarrhea (5–10 h)	less than 100%
-dehydration/only oral hydration	not more than 1/3 of children

* OFC—oral food challenge; ** 3 children dropped out of the study.

**Table 3:** Symptoms of allergy to new foods introduced into the diet in the study group of children with chronic food protein-induced enterocolitis syndrome (FPIES) dependent on cow’s milk protein intake.

Characteristic	Number of Children (*n* = 55)
Foods that triggered IgE-independent allergy symptoms in infants after new food intake *:	6 children (11%)
-apple	2 children (3.6%)
-rice	2 children (3.6%)
-chicken meat	1 child (1.8%)
-turkey meat	1 child (1.8%)
Symptoms of IgE-independent allergy after new food intake *:	
-skin lesions **	4 foods (11%)
-diarrhea	2 foods (3.6%)
Foods that triggered IgE-dependent allergy symptoms (mild urticarial) in infants after new food intake:	2 children (3.6%)
-egg white	1 child (1.8%)
-wheat	1 child (1.8%)
Foods that triggered IgE-dependent allergy symptoms (extensive urticarial) in the next years after new food intake:	3 children (5.5%)
-peanuts	2 children (3.6%)
-soya	1 child (1.8%)

* The provocation was performed twice; ** looked like atopic dermatitis but without itching.

**Table 4:** Introducing new foods to the diet in the study group of children with chronic food protein-induced enterocolitis syndrome (FPIES) dependent on cow’s milk protein intake.

Characteristic	Foods
Age of onset of new foods in the diet:	
-after 4 months	89% of children
-after 5 months	11% of children
Vegetables to be introduced to the diet at 4–6 months of age:	Pumpkin, broccoli, cauliflower then carrots, potatoes, green beans, beets, zucchini Parsley
Fruits to be introduced to the diet after 5 months of age	Peach, grapes, avocado, watermelon, blueberries
Foods to be introduced to the diet after 6 months of age:	
-vegetables	Sweet potatoes and green peas
-fruits	Apple, pear, banana, strawberries
-cereals	Corn, wheat
-meats	Rabbit meat, pork, then turkey meat
-fish	Cod, salmon, and others
Eggs to be introduced to the diet after 7 months of age	First yolk, then egg white
Cereals to be introduced to the diet after 8 months of age	Rice, oats
Meats to be introduced to the diet after 8 months of age	Beef, chicken meat
Foods to be introduced to the diet after 12 months of age	Soya, peanuts

## Data Availability

The results of the tests are included in the records of the clinics where the children were treated.
